# Characteristic patterns of EEG oscillations in sheep (*Ovis aries)* induced by ketamine may explain the psychotropic effects seen in humans

**DOI:** 10.1038/s41598-020-66023-8

**Published:** 2020-06-11

**Authors:** A. U. Nicol, A. J. Morton

**Affiliations:** Department of Physiology, Development and Neuroscience, University of Cambridge, Downing Street, Cambridge, CB2 3DY UK

**Keywords:** Neuroscience, Physiology

## Abstract

Ketamine is a valuable anaesthetic and analgesic that in recent years has gained notoriety as a recreational drug. Recently, ketamine has also been proposed as a novel treatment for depression and post-traumatic stress disorder. Beyond its anaesthetic actions, however, the effects of ketamine on brain activity have rarely been probed. Here we examined the cortical electroencephalography (EEG) response to ketamine of 12 sheep. Following ketamine administration, EEG changes were immediate and widespread, affecting the full extent of the EEG frequency spectrum measured (0–125 Hz). After recovery from sedation during which low frequency activity dominated, the EEG was characterised by short periods (2–3 s) of alternating low (<14 Hz) and high (>35 Hz) frequency oscillation. This alternating EEG rhythm phase is likely to underlie the dissociative actions of ketamine, since it is during this phase that ketamine users report hallucinations. At the highest intravenous dose used (24 mg/kg), in 5/6 sheep we observed a novel effect of ketamine, namely the complete cessation of cortical EEG activity. This persisted for up to several minutes, after which cortical activity resumed. This phenomenon is likely to explain the ‘k-hole’, a state of oblivion likened to a near death experience that is keenly sought by ketamine abusers.

## Introduction

Ketamine is a neuroactive phencyclidine derivative highly valued for its anaesthetic and analgesic properties^1,2^. Used in both medicine^3^ and veterinary medicine^4^ it has a rapid onset of action and does not cause respiratory depression^5^. Termed a ‘dissociative’ anaesthetic, it causes a characteristic anaesthesia whereby patients are catatonic and do not appear to process information, yet can swallow and open their eyes^3^. The analgesic effects of ketamine occur at lower concentrations than its anaesthetic effects, and persist beyond emergence from anaesthesia^6^. Ketamine is particularly valuable as an anaesthetic and analgesic where resources are limited, such as in field situations and developing countries. These qualities have earned ketamine its place on the World Health Organisation’s essential drug list^7^.

The dissociative state induced by ketamine almost certainly underlies its appeal as a recreational drug^8^. Psychic sensations associated with emergence from ketamine anaesthesia are frequently reported, and because of this, clinical use of ketamine is largely restricted to veterinary medicine and young or elderly human patients^9^. Subjective effects include perceptual distortions, sensations of floating, vivid dreams or illusions, distortion of sense of time and space, and alterations in mood state and body awareness^10^. At a sufficiently high dose, both awareness of self and surroundings, and interactions with others become profoundly impaired - a state known as the ‘k-hole’^11^. Dissociation of the conscious from the physical self under the influence of ketamine is substantiated in psychophysiological studies in normal subjects (e.g. the false hand illusion^12^).

In addition to its well-described uses in the fields of anaesthesia, analgesia and drug abuse, ketamine has emerging applications in two other fields. Although it has not been implicated in long-term psychotic reactions in normal subjects, ketamine has been of interest in schizophrenia research for many years because it can precipitate the emergence of psychosis in schizophrenic patients^10^. It is being used to develop animal models of schizophrenia^13^. Clinically, ketamine has attracted much recent attention following reports of its efficacy in treating psychiatric disorders, particularly depression^14^ and post-traumatic stress disorder^15^. Nevertheless, despite the increased interest in ketamine as a tool or treatment, little is known about its non-anaesthetic effects on brain function.

Here we investigated directly the effects of ketamine on cortical EEG activity using normal sheep with chronically-implanted subdural recording electrodes. We chose to use sheep for this study, because ketamine is commonly used as a large animal anaesthetic^6^, and its clinical (anaesthetic and analgesic) effects and pharmacokinetics are well understood^16^. In recent years, sheep have been recognised as a species that is eminently suitable for use as pre-clinical models of human neurological disorders^17,18^. They have a large complex brain with a gyrencephalic cerebral cortex and basal ganglia that are anatomically similar to those of non-human primates. They are physically robust, and their skull anatomy is well-suited to bearing chronically implanted cranial devices^19,20^. Importantly, they are intelligent and trainable yet docile animals that are easy to manage safely in the laboratory^20^. These characteristics make them very suitable for studying the effects of neuroactive drugs, including anaesthetics.

Over a period of several months, we explored the EEG response at different doses of ketamine, up to 24 mg/kg (a high anaesthetic dose at the lower end of the range used recreationally). In addition to identifying the EEG signatures that characterize the sedative effects of ketamine, we identified two phenomena that may explain the psychic experiences associated with ketamine. The first is a distinct and characteristic alternating oscillatory state, whereby the output of the whole cortex switches between co-ordinated bursts of low and high frequency oscillations. The timing of this phenomenon makes it likely to underlie the dissociative state. The second, previously undescribed, phenomenon was the temporary but complete cessation of EEG cortical activity induced by ketamine. This phenomenon is likely to explain the ‘k-hole’ described and keenly sought by ketamine abusers^11^.

## Results

### Characteristic dose-dependent behavioural and quantitative EEG responses of sheep to ketamine

**Figure 1:**
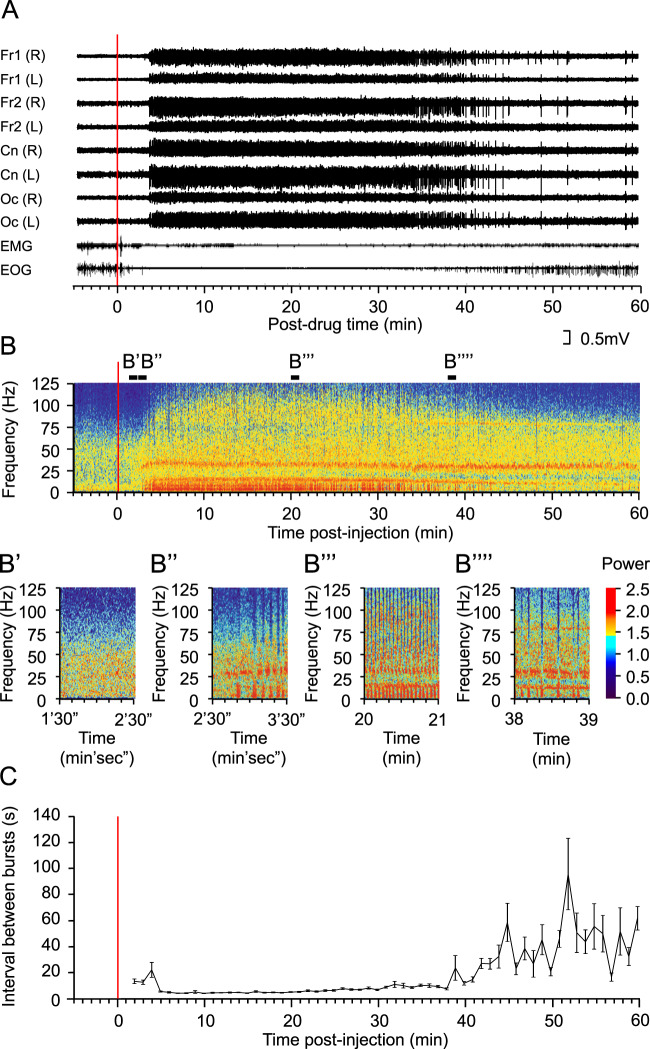
The EEG response to ketamine. (**A**) Cortical EEG data were recorded via 8 electrodes positioned over occipital (Oc), central (Cn) and two frontal (Fr1, Fr2) areas of both left and right hemispheres (L, R). Electromyographic (EMG) data were recorded from electrodes in the left and right dorsal neck muscles and electrooculography (EOG) from periorbital electrodes. The trace shows the differential signal between the two electrodes for the EMG and between electrodes positioned anterior and posterior to the left eye for the EOG. (**B**) The left occipital EEG is shown as a power spectrogram. Four sections of the spectrogram are expanded in B’ – B””. (**C**) The mean (± s.e.m.) interval between low frequency bursts for each minute following intramuscular drug delivery is shown (n = 5 sheep). Intramuscular ketamine (12 mg/kg) delivery started at the red line in A-C. The key in B shows the pseudocolour range of power (mV^2^).

The behavioural response of sheep to ketamine can be separated into three distinct sequential phases based on the behaviour and the electromyogram (EMG)/electroocculogram (EOG) activity (Fig. 1). The first phase, seen shortly after delivery of ketamine, is characteristic of the well-described anaesthetic effect^21^. During this phase, voluntary movement is suppressed, as are eye movements, although the eyes remain open and they have intact palpebral (eye blink) reflexes. In this phase there is typically increased muscle tone^22^, which was evident in the EMG trace (see below). In the second phase, sheep are conscious and respond to gentle sensory stimulation such as incidental noise or movements in the visual field, but do not engage in voluntary movement. This corresponds to the dissociative analgesic phase. During the third phase, sheep are conscious and appear alert, with awake levels of EMG and EOG activity, although again they do not engage in voluntary movement. The duration of each of these phases is dose-dependent (data not shown).

**Figure 2:**
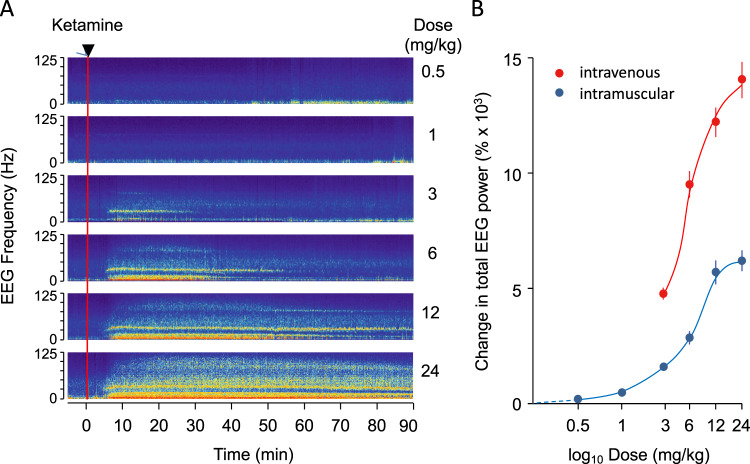
The dose-response to ketamine. Spectrograms in **A** show the power across the frequency spectrum of the EEG (using the same pseudocolour key shown in Fig. 1B) recorded from the left frontal cortical electrode in a single sheep when ketamine was given (red line) intramuscularly at each of 6 doses (0.5–24 mg/kg). These data were collected in six separate experimental sessions, one for each dose. (**B**) The mean (±s.e.m.) change in total power across the EEG spectrum (0–125 Hz) measured in the first 15 min post-injection relative to that of a 10 min pre-injection baseline period is plotted against dose for sheep receiving intramuscular (N = 5; blue symbols) or intravenous (N = 7; red symbols) ketamine.

The effect of ketamine on the EEG was similar for all recording channels (see Fig. 1A for an example response to 12 mg/kg ketamine). The latency to onset and the total power of the EEG response were dose- and route-dependent (Fig. 2; Supporting Information (SI) Table S1). Differential pharmacological effects can be seen clearly when separate frequency bands are analysed after drug delivery (Fig. 2A; SI Fig. S1; SI Tables S1, S2). A quantitative EEG (qEEG) analysis was conducted for all doses using either total power (0–125 Hz; Figs. 1B and 2A), or classically defined frequency bands of delta (0–4 Hz), theta (4–9 Hz), alpha (9–14 Hz), beta (14–35 Hz) and gamma (>35 Hz). Repeated measures ANOVAs were performed for response latency and half-life in which different frequency bands were treated as repeated measures. Full factorial analyses incorporated the hemisphere and region as within subjects factors. In neither analysis were there any significant interactions involving either of these factors. In response to 12 mg/kg of ketamine, the earliest changes in power were seen in the low frequency (<14 Hz) ranges and these subsided most rapidly. By contrast, at 12 mg/kg the increased power in the gamma bands started significantly later (F_1,10_ = 32.8, P < 0.001) and persisted significantly longer than those in the lower frequency bands (F_1,10_ = 13.0, P = 0.005). Both beta and gamma power remained elevated for more than 1 h after administration, regardless of the route of drug delivery (SI Fig. S1, Table S1). The spectral profile of the response also varied according to dose, particularly in the high gamma range. The half-lives of the slow frequency band oscillation changes correlated closely with the duration of sedation (behavioural phase 1; SI Table S2).

### Ketamine induces alternating bursts of theta-gamma oscillations that correlate with the dissociative phase

**Figure 3:**
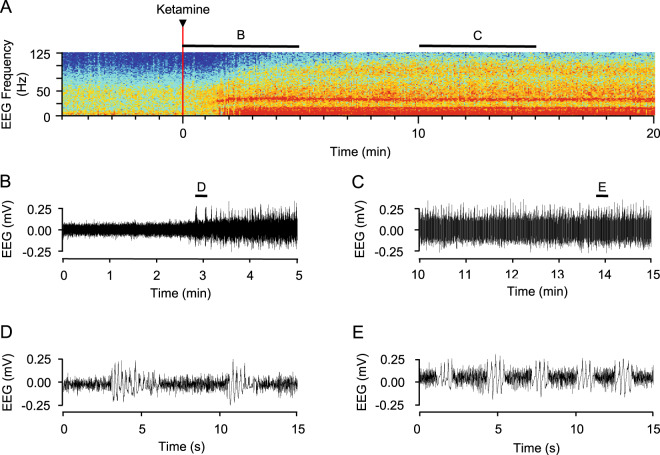
Ketamine-evoked alternation between high and low frequency EEG oscillations. EEG activity recorded from a single electrode (left frontal) after intramuscular delivery of 12 mg/kg ketamine (red line) is shown as a spectrogram (**A**). The key to the pseudocolour is shown in Fig. 1B. In **B** and **C**, the EEG traces are shown for the periods indicated by the black bars above the spectrogram in **A**. The position in time of the EEG traces in **D**, **E **are shown as black bars in **B** and **C** respectively.

In the second behavioural phase, when animals are responsive but do not engage in voluntary activity, the EEG was remarkable for a spectral phenomenon observable in both the raw EEG trace (Fig. 3) and the total power spectrograms (Figs. 1B and 3A). During this phase, a characteristic pattern of oscillations emerged, in which bursts of slow wave activity coupled with silence in the gamma range alternated with bursts of gamma oscillations with silence in the slow wave range. The bursts were irregular at first (Figs. 1B” and 3B,D) but regularised within a few minutes of drug delivery (Fig. 1B” and 3C,E). Once established, this pattern of activity took the form of ~1–2 s bursts of slow frequency oscillations alternating with ~2–3 s of gamma oscillation (Fig. 3E, SI Fig. S2). This dominated the spectral profile and persisted for >30 minutes (see for example the response to a 12 mg/kg dose; Fig. 1C). Waveform correlation analysis showed that bursts of oscillation in both the lower (delta, theta and alpha) and high frequency (gamma) ranges were synchronised in phase (SI Fig. S2). The bursts of low frequency oscillations were synchronised in anti-phase with the bursts of high frequency oscillations. Beta frequency oscillations (14–35 Hz) had the least tendency to occur in bursts, and there was only a weak correlation between beta activity and either low or high frequency burst activity.

### Total cessation of EEG activity after intravenous ketamine

**Figure 4:**
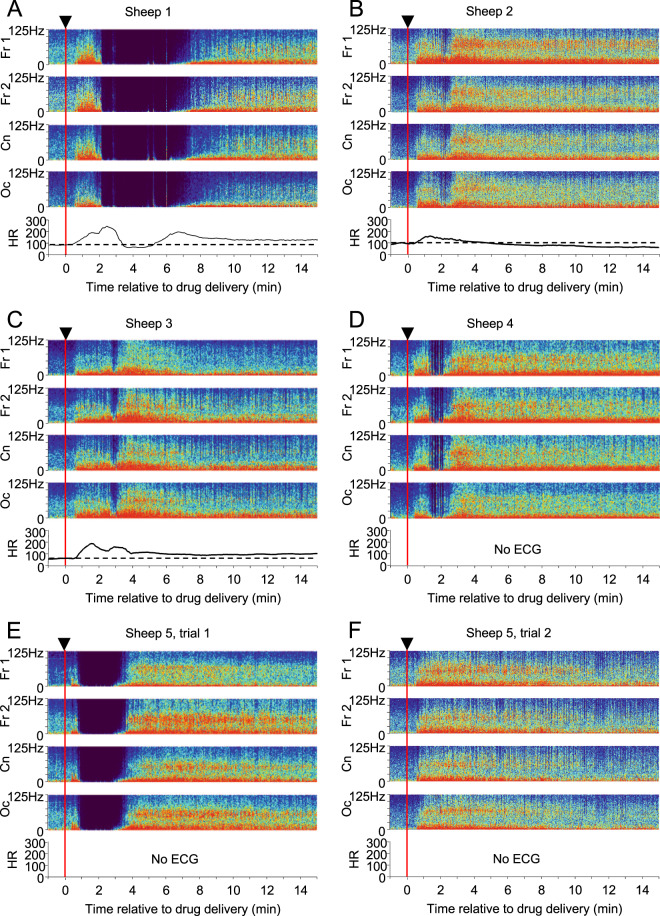
EEG holes are visible in spectrograms after intravenous ketamine delivery. (**A–F**) Spectrograms of EEGs recorded from 5 sheep that received i.v. ketamine (24 mg/kg; red line in A-F). For 3 of these sheep (**A–C**) the heart rate (HR) was recorded via ECG. Sheep 5 (**E**) received a repeated dose of ketamine in a separate session 3 days after the first trial (**F**).

After the highest dose of intravenous ketamine (24 mg/kg), a second EEG phenomenon was observed that has never been described before (Fig. 4). Initially, the characteristic response to ketamine was seen. Within 2 minutes of drug delivery, however, EEG activity ceased completely in 5/6 sheep. We term this phenomenon an ‘EEG hole’. The extent and duration of this phenomenon varied between sheep. For 2 sheep, the interruption in the response was total and sustained for several minutes (Fig. 4A,E). For the other 3 sheep, the interruption in the EEG was shorter, and punctuated by intermittent brief periods of resumed EEG activity (Fig. 4B–D). When it occurred, the profile of the EEG hole was similar for all recording electrodes across the brain (SI Fig. S3). Shortly before onset of the hole there was an increase in muscle tone in the EMG recorded from the neck muscles (SI Fig. S4). This was evident in each case when the EEG hole occurred, and was additional to the already increased EMG activity which is characteristic in ketamine anaesthesia^22^. A similar increase in tone was also apparent in EOG activity during the hole. During the period when the EEG holes were recorded, all sheep appeared to be deeply sedated, and there was no overt indication of any unusual brain activity. On resumption of EEG activity, a typical phase 2 response to ketamine was evident. That is, the pattern of activity that returned was the alternating bursts of low and high frequency activity in the EEG that would be expected at that time after ketamine administration. Interestingly, one of the sheep in which this phenomenon was seen (sheep 5, Fig. 4E) received a repeat of this dose of ketamine in a separate trial three days later. After the second trial, no EEG hole was seen (Fig. 4F).

The behaviour of the sheep during the EEG hole was unremarkable. They looked sedated, but otherwise did not show any adverse effects. In fact, for sheep 2 and 3 (Fig. 4B,C), the dropout of cortical activity was not observed during the recording period but was found only when the spectrograms were constructed. For some sheep, when recordings were made at 24 mg/kg ketamine, we added an extra recording channel to collect ECG. As reported elsewhere^5^, ketamine delivery increased the heart rate (Fig. 4A–C). In the sheep when the EEG hole lasted for several minutes (Fig. 4A), the heart rate fell to the pre-drug resting rate during the quiescent period. When cortical activity recovered, the heart rate returned to an elevated rate.

## Discussion

Ketamine is highly valued for its anaesthetic and analgesic properties, yet few studies have examined in detail the EEG response to this drug. Here, we used sheep with chronically implanted electrodes to study the effect of ketamine on cortical EEG. The EEG response to ketamine showed a characterised increase in EEG power, as has been described previously. A high resolution analysis of the EEG, however, revealed that the changes were more complex than a simple increase in power. On emergence from the sedation phase the EEG was characterised by 3–4 s of high power, high frequency bursts that alternated with 1–2 s of high power low frequency bursts. While the subjective experience of sheep cannot be determined, the clinical and psychic profile of ketamine administration is very well described in humans, and the timing makes it likely that this ‘oscillation of oscillations’ underlies the dissociative state caused by ketamine. A second striking finding was the occurrence of holes in the EEG activity at an intravenous (i.v.) dose of ketamine in the low recreational range^23^. To our knowledge, this is the first report of such an effect. It seems likely that the total cessation of cortical activity underpins the phenomenon known as the ‘k-hole’. This is a state of oblivion keenly sought by recreational users that is associated with an hallucinogenic experience on emergence from this state, and likened to near-death experience^11^.

The cortical response to ketamine of sheep appears to be similar to that of humans. Relatively few EEG studies have been made of humans under the influence of ketamine, and all of those have been conducted over short periods, using lower doses of ketamine, often in adjunct with other anaesthetics^24,25^. Nevertheless, the spectrograms we generated after ketamine administration in sheep resembled those of human subjects receiving intravenous ketamine as a general anaesthesia^25,26^. The EEG oscillation of oscillations we saw in the sheep also matched the description of the EEG during the catatonic state in ketamine-anaesthetised children^24^.

In this study, the different doses of ketamine were administered in the same order for each sheep. This was done to minimise potential hangover effects by ensuring that higher doses followed on from lower doses. This is a limitation of the study, as it potentially introduced a cumulative bias into the data. However, we think this is unlikely to be a large effect, since in the one case where the same high dose was repeated, there was desensitisation, rather than amplification, of the response to the second dose.

Better awareness of the effects of ketamine on the brain would greatly aid our understanding of its use, not only as an anaesthetic, analgesic or antidepressant but also as a drug of abuse. Human volunteer studies of anaesthetic action are invaluable, but such studies are challenging to conduct as well as risky^27^. Since the oscillatory changes we describe in the sheep have been reported in humans but not in rats or mice, rodents may not be ideal species for translational research into the action of ketamine. Sheep could bridge this translational gap. While not commonly used for neuropharmacological studies, they are a valuable model species for studying the brain^19,28^. The brain of the sheep is highly developed, with a thick and well-developed cerebral cortex similar to that seen in primates. We have made successful EEG recordings from sheep with subdural electrodes that have lasted more than 3 years. The inter-subject variability due to natural variation in brain anatomy and/or electrode placement is offset by high intra-subject stability. A group of 5–6 sheep is sufficiently powered to see small (<10%) changes in EEG responsiveness if a study is conducted longitudinally. Finally, although sheep are not currently used as animal models of psychiatric conditions they would make excellent models for psychiatric illnesses^29^, since they have emotional responses to the stressors that cause depression and anxiety in humans^18,29^.

The precise physiological mechanisms through which ketamine exerts its effects are unknown. Disruption of cortical network connectivity is believed to underlie ketamine anaesthesia^30^, and probably forms the basis for the hallucinogenic sensations sought by recreational users of the drug^8^. The ketamine-induced alternation of high and low frequency oscillations seen across the cortex adds a new challenge to understanding the network connectivity during ketamine administration, since they occur on a time scale that is below the resolution of most functional magnetic resonance imaging studies^26^. Both high and low frequency cortical oscillations are thought to be important for network function, with elevated power in high frequency oscillations promoting local connectivity and elevated power in low frequency oscillations favouring connectivity across wider cortical distances^31^. The alternating partitioning of these two activities may explain, at least in part, the dissociative nature of the effects of ketamine. Although local network activity may be enhanced with increased high frequency oscillations, the temporal dissociation between the high and low frequency components of the EEG is likely to reduce functional networking between cortical regions. This may be of particular relevance to studies of schizophrenia, where disrupted cortical networks are thought to underlie, at least in part, some of the symptoms. Understanding how different brain regions engage and disengage is key to understanding the function of neural networks. Ketamine-evoked changes in the EEG provide an interesting tool for studying such networks, not only in the normal brain but also in neurological diseases in which cognitive and psychiatric disorder are prominent.

## Methods

### Animals

The sheep used in this study were locally sourced Welsh mountain ewes (n = 5) and merino ewes (n = 7). All of the sheep were neurologically and genetically normal. All procedures involving the Welsh mountain sheep were conducted at the University of Cambridge in accordance with the UK Animals (Scientific Procedures) Act (1986), and the University of Cambridge ethical review board. The Welsh mountain sheep were reared in Cambridge, UK. At surgery their age was approximately 2 years and 3 months, and they weighed 46 ± 3.2 kg (SEM). All procedures involving the merino sheep were conducted at the Preclinical Imaging and Research Laboratories (PIRL) of the South Australian Health and Medical Research Institute (SAHMRI) and followed the requirements of the SAHMRI Animal Ethics Committee including the Australian Code for the Care and Use of Animals for Scientific Purposes (8^th^ Edition 2013). These sheep were part of a group that included transgenic animals. Accordingly, although the sheep that were used in this study were genetically normal, all handling of these sheep conformed to physical containment conditions as approved by the Institutional Biosafety Committee and the Office of the Gene Technology Regulator (OGTR, Australia). At surgery the merino sheep were approximately 5 years of age, and their weight was 85 ± 2.5 kg.

### EEG implantation surgery

Surgical and anaesthetic procedures are described in detail in Perentos *et al*.^19,20^. Briefly, anaesthesia was induced using alfaxalone (3 mg/kg; Alfaxan®, Jurox, U.K., i.v.) for the Welsh mountain sheep, and diazepam (0.4 mg/kg) and ketamine (5 mg/kg, i.v., Ketaset, Zoetis Inc., New Jersey, USA) for the merino sheep. The upper airway was intubated with an endotracheal tube, and general anaesthesia was maintained during surgery with isoflurane in oxygen and nitrous oxide for the Welsh mountain sheep and isoflurane in oxygen for the merino sheep. Isoflurane was maintained at 2–3%, end-tidal CO_2_ at 25–30 mmHg and mean arterial blood pressure at 70–90 mmHg. Intravenous fluids were supplied at a rate of 5 ml/kg/h (lactated Ringers, Hartmann’s Solution 11 by Aquapharm). Vital functions were recorded at 5 min intervals, and blood gases sampled every 30 min throughout the procedure.

Once general anaesthesia was achieved, the sheep was positioned in sternal recumbency for surgery. The head was fixed in a stereotaxic frame (Kopf Instruments, USA). An incision was made at the midline between the eyes, and extended to the external occipital crest of the skull. The scalp was retracted using blunt dissection. The periosteum and all traces of connective tissue and fat were cleared from the skull, allowing visualisation of the bregma, here defined as the point of intersection of the midline scull suture and the transverse suture between the frontal and parietal bones. Electrode positions are defined relative to the position of the bregma. Subdural electrodes (3 mm diam. × 1 mm deep Ag/AgCl disc, NDimension (Science and Engineering) Ltd., Cambridge, UK) were implanted via craniotomies ~25 mm, ~15 mm and ~5 mm anterior, and ~10 mm posterior to bregma, and 10 mm lateral to the midline over both hemispheres. A reference electrode was inserted at the midline 10 mm posterior to bregma, and 2 ground wires were attached to the skull using stainless steel screws. Stainless steel coil electrodes were implanted bilaterally in the dorsal splenius muscles of the neck for recording EMG data, and Ag/AgCl electrodes were positioned in the inner and outer canthi of both eyes for recording the EOG. When all electrodes and wires were in place, a rigid cap was formed using dental acrylic containing Gentamicin (DePuy, Johnson & Johnson) to seal all of the components and craniotomies. Leads from all electrodes terminated at a multi-pin connector (Omnetics Connector Corporation, MN, USA). The connector was either exteriorised near the occipital crest (1 sheep), or housed in a 3D-printed polyamide (nylon) chamber (G.E. Baker (UK) Ltd) fixed to the skull. The chamber had a screw cap that allowed easy access to the connector post-surgery.

During surgery, a non-steroidal anti-inflammatory (carprofen) was administered. To manage possible post-operative infection, pain and/or inflammation, all sheep received buprenorphine at the end of surgery, and daily carprophen and antibiotics for three days after surgery. If necessary, additional buprenorphine was administered in the first three days according to veterinary instruction.

Recordings from the Welsh mountain sheep in this study were conducted 1–10 months post-surgery. Those from the merino sheep took place 6–10 months post-surgery.

### Electrophysiological recording

EEG data were collected wirelessly using a MCS advanced wireless system (Multichannel Systems Gmbh, Germany). At the commencement of each recording session, each sheep was fitted with a 16 channel wireless head-stage (W2100-HS16). Recordings could be made simultaneously from up to 8 sheep at a time. Data were collected at a sampling frequency of 1 kHz on each channel.

### Ketamine administration and recordings

Ketamine was delivered to individual sheep suspended in veterinary slings with their hooves elevated off the ground. Sheep quickly became accustomed to being restrained in this way, enabling a baseline recording period of 10–15 minutes preceding ketamine delivery. Recordings were typically made from 2–4 sheep at a time.

For the Welsh mountain sheep, ketamine was delivered intramuscularly as a single injection to the gluteus muscle (left or right). Merinos received ketamine intravenously via a jugular catheter. Catheters were fitted at least 24 h before ketamine treatment. To ensure patency, the catheters were flushed daily with heparinised saline. Ketamine injections were delivered between 10:00 and 16:00.

Ketamine (100 mg/ml ketamine hydrochloride solution; Ketaset, Zoetis Inc., New Jersey, USA) was administered when the sheep had remained calm in the sling for a minimum period of 15 min. All sheep received a separate dose of 3, 6, 12 and 24 mg/kg body weight. The doses were given in the same order to all the sheep so that any possible hangover effects would be similar for all animals. The order was 6, 12, 3, 24 mg/kg, with at least 2 days between doses. One merino sheep received a second dose of 24 mg/kg 3 days after the first. In this case, while both recordings are described here, only the second recording was included in subsequent statistical analysis. All of the Welsh mountain sheep received two additional intramuscular doses of 0.5 and 1 mg/kg ketamine.

Drug trials were performed in separate recording sessions with a minimum of 24 h between doses. During recording after ketamine delivery (when two operatives were present), the sheep remained undisturbed (with a single observer present) for a minimum period of 1 h, but otherwise as long as was necessary. When they were responsive and deemed competent to stand and move normally, they were returned to their home pen. This was never greater than 90 min after the administration of ketamine.

### Electroencephalography analysis

EEG data were down-sampled to 250 Hz to promote efficient data processing. In subsequent analyses, EEG data were considered in terms of the contribution of identified frequency bands to the overall frequency spectrum. These frequency bands reflect oscillatory activity recognised as delta (0–4 Hz), theta (4–9 Hz), alpha (9–14 Hz), beta (14–35 Hz) and gamma (35–125 Hz). The theta and gamma bands were further sub-divided into low and high theta (4–6 Hz, 6–9 Hz respectively), and into low, mid and high gamma (35–55 Hz, 55–85 Hz, 85–125 Hz respectively).

In an initial pre-processing stage, a global reference was generated for each sheep by computing the instantaneous average of all channels of EEG data. This global reference was then subtracted from each channel of EEG data. Thus, each EEG channel was represented as a differential signal relative to the global reference. This stage was conducted to minimise contamination of the EEG data by variation at the reference electrode.

The power of each oscillatory frequency in the EEG was computed using a fast Fourier transform (FFT). The power spectrum generated by this analysis reflects the contribution of each frequency to the total power of the EEG. For each recording, a temporal representation of the power spectrogram was produced in the form of a spectrogram. In the spectrograms, EEG power as a function of mV^2^ is displayed according to the colour spectrum in the key in Fig. 1B. In some analyses, the root mean square (RMS) of the EEG was computed. In this measure, the mean amplitude of the signal was computed in 1 s windows. This value was then squared and the square root of the resulting value calculated, thereby providing a finite positive measure of EEG activity.

Latencies of responses induced by ketamine delivery were computed for each EEG band as the time from start of ketamine delivery to the point at which the power in a given band increased by 100% relative to the stable pre-drug baseline. This was also computed for the change in total power across the full frequency spectrum (0–125 Hz).

Waveform correlation analysis was performed for certain comparisons between certain traces. This measures the similarity of two waveforms. Starting with the two channels aligned, each data point amplitude in the reference waveform is multiplied by each data point amplitude in the other, and summing the results. The reference waveform is then advanced by one sample point and the process repeated until all points in the reference channel are accommodated. In the resulting correlation waveform, a symmetrical upward peak implies that the two waveforms are synchronised and in phase, while an inverted peak implies that the two waveforms are in anti-phase.

### Electromyographic recordings

EOG and EMG traces were down-sampled to 250 Hz to promote efficient analysis. For both channels of EMG data, an average reference was computed as the average of the two EMG channels and all EEG channels. This average reference was then subtracted from each channel of EMG data. This was found to provide the most stable representation of muscle activity. A single EMG channel was then computed as the differential between the two average-referenced EMG channels. For the EOG data, a global reference was computed as the average of all four EOG channels. This average reference was then subtracted from each individual channel of EOG data. For certain analyses the EMG and EOG data were RMS transformed, as described above for the EEG, as a measure of absolute muscle activity.

### Electrocardiogram recordings

In some of the trials with intravenous ketamine the electrocardiogram (ECG) was recorded. These recordings were made for the merino sheep implanted in the second surgery session. This was achieved by attaching a single self-adhesive ECG patch to the thorax next to the left forelimb. This area is relatively free from wool and provides a good surface from which to acquire these data. During acquisition the ECG data were referenced to the same intracerebral reference as the other electrophysiological data. During offline processing an average reference was computed as the average of the ECG signal and all of the EEG channels, and this was subtracted from the ECG signal.

### Statistical analyses

Repeated measures analyses of variance (ANOVA) were performed using SPSS (IBM Corp., Released 2016, IBM SPSS Statistics for Windows, Version 24.0, Armonk, NY: IBM Corp.) with different EEG frequency ranges entered as a within subjects variable. These analyses incorporated between subjects factors sufficient to account for each data instance: dose, trial, route of administration, hemisphere (left/right), brain region. In these analyses, data were pooled across factors which yielded no significant effect or interactions in full factorial analysis. Statistical significance was defined as *P* < 0.05.

## Supplementary information

Supplementary information.
