# Supplementary material for: Characteristic patterns of EEG oscillations in sheep (Ovis aries) induced by ketamine may explain the psychotropic effects seen in humans
Source: Sci Rep. 2020 Jun 11;10:9440. doi: 10.1038/s41598-020-66023-8 (PMC7289807; doi:10.1038/s41598-020-66023-8)
Supplement: Supplementary file 1 — Supplementary information. [file 41598_2020_66023_MOESM1_ESM.pdf]

Characteristic patterns of EEG oscillations in sheep (*Ovis aries*) induced by ketamine may explain the psychotropic effects seen in humans

AU Nicol and AJ Morton

Department of Physiology, Development and Neuroscience, University of Cambridge, Downing Street, Cambridge CB2 3DY, UK

Author for correspondence:

Prof AJ Morton
Department of Physiology, Development and Neuroscience
University of Cambridge
Downing Street
Cambridge CB2 3DY, UNITED KINGDOM

Email ajm41@cam.ac.uk

Supplementary Information Figure S1.

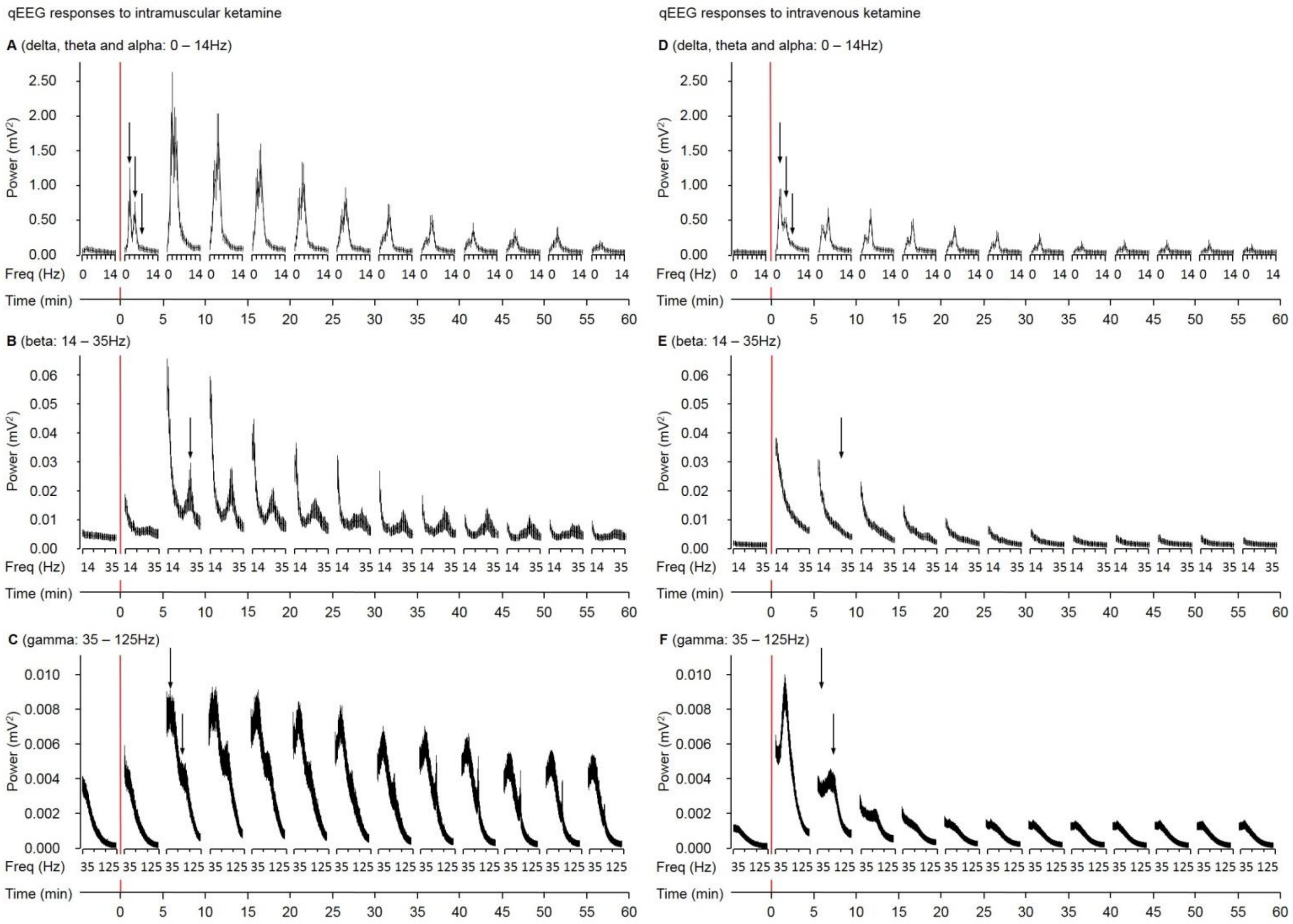

Supplementary Information Figure S1. Ketamine-evoked changes in the EEG power spectrum.

Fast Fourier transform analysis was conducted to show separate frequency power spectra for the EEG data. Here the mean (± s.e.m.) power is shown for sheep that received ketamine at 12mg/kg either intramuscularly (i.m., **A-C**, n = 5) or intravenously (i.v., **D-F**, n = 7) in 5 min windows. Data are displayed for the low (**A, D**; 0 – 14Hz), medium (**B, E**; 14 – 35 Hz) and high frequency (**C, F**; 35 – 125 Hz) components of the EEG. The downward arrows in A-F indicate six peaks distinguishable in the power spectrum in EEG data recorded after either i.m. or i.v. administration. These occur in bands identifiable as delta, low and high theta, beta, and gamma.

The differential pharmacological effects can be seen clearly when separate frequency bands are analysed after i.v. or i.m. drug delivery (see also SI Fig. S2; SI Table S2). For example, in response to 12mg/kg i.v. of ketamine, the largest and earliest changes in power were seen in the low frequency (<14Hz) ranges and these subsided most rapidly. By contrast, at the same dose the increase in power in gamma bands started later ($F_{1,10}$=32.8, P<0.001) and persisted significantly longer than those in the lower frequency bands ($F_{1,10}$=13.0, P=0.005). These ANOVAs were performed on repeated measures data for 12mg/kg that were pooled across hemisphere and brain region as these factors yielded no significant effect or interactions. Both beta and gamma power remained significantly elevated for more than 1 h after administration,

regardless of the route of drug delivery. The half-life of the slow frequency band oscillation changes correlated directly with duration of sedation (behavioural phase 1; SI Table S2).

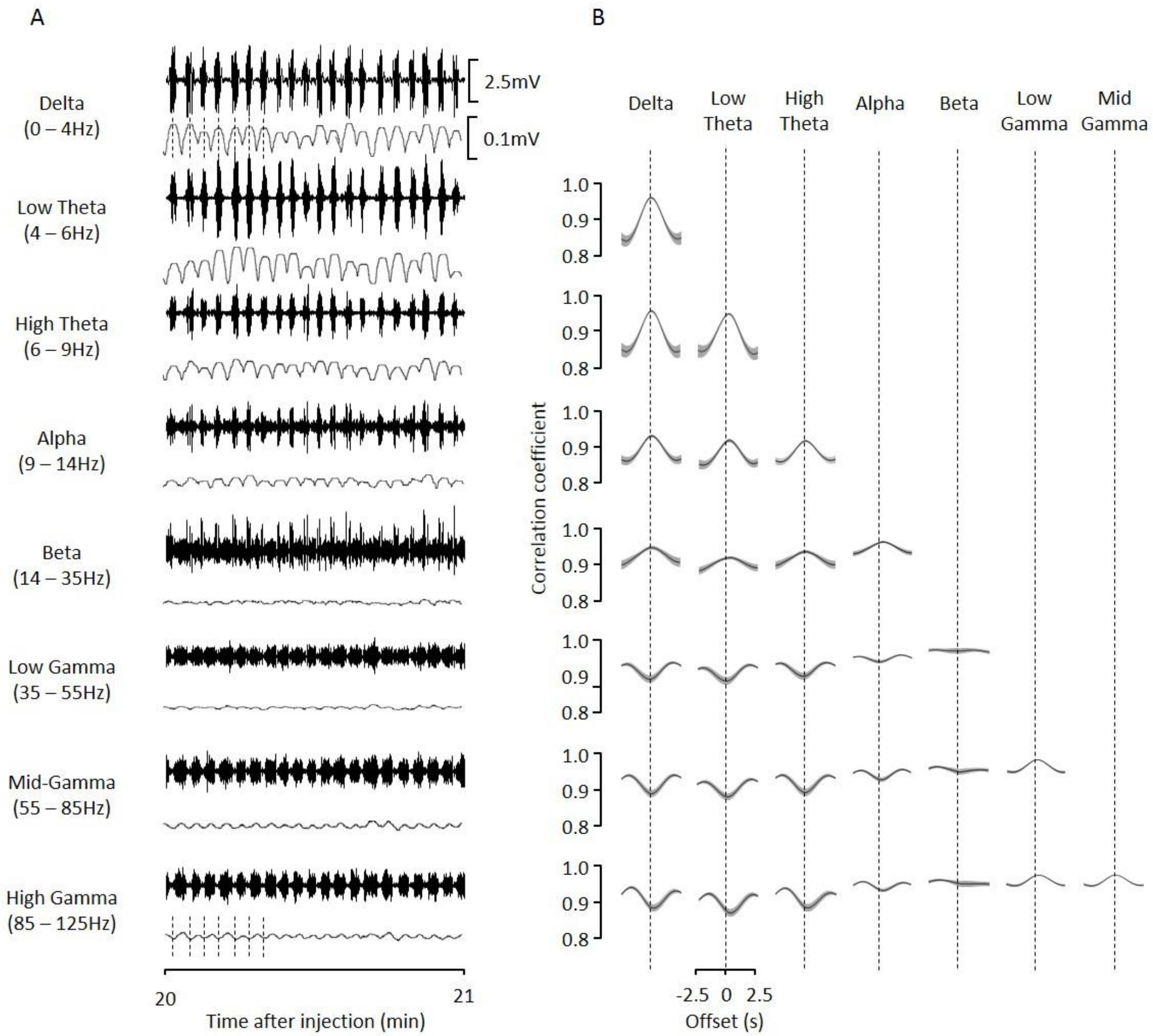

Supplementary Information Figure S2. Alternating bursts of high and low frequency EEG activity.

EEG traces in **A** show the recording corresponding to the spectral data shown in Fig 3. To examine the temporal relationship between bursts of different frequencies in behavioural phase 2, each frequency range was band-pass filtered and then root mean

square (RMS)-transformed, with a 1 s time constant. The RMS transformed data for each frequency range are shown underneath each recording trace. Waveform correlations were computed with a 5 s processing window for each pairwise combination of transformed traces and in **B** the mean correlations (± s.e.m. shown as grey shading) from EEG of sheep that received i.m. ketamine at 12 mg/kg (n = 5) are shown. The strength of correlation reflects the likelihood that activity in one trace is associated with activity in another trace at a given point in time; so upwards deflections with zero offset indicates in-phase activity, whilst downwards deflection at zero offset indicates antiphase activity.

Supplementary Information Figure S3.

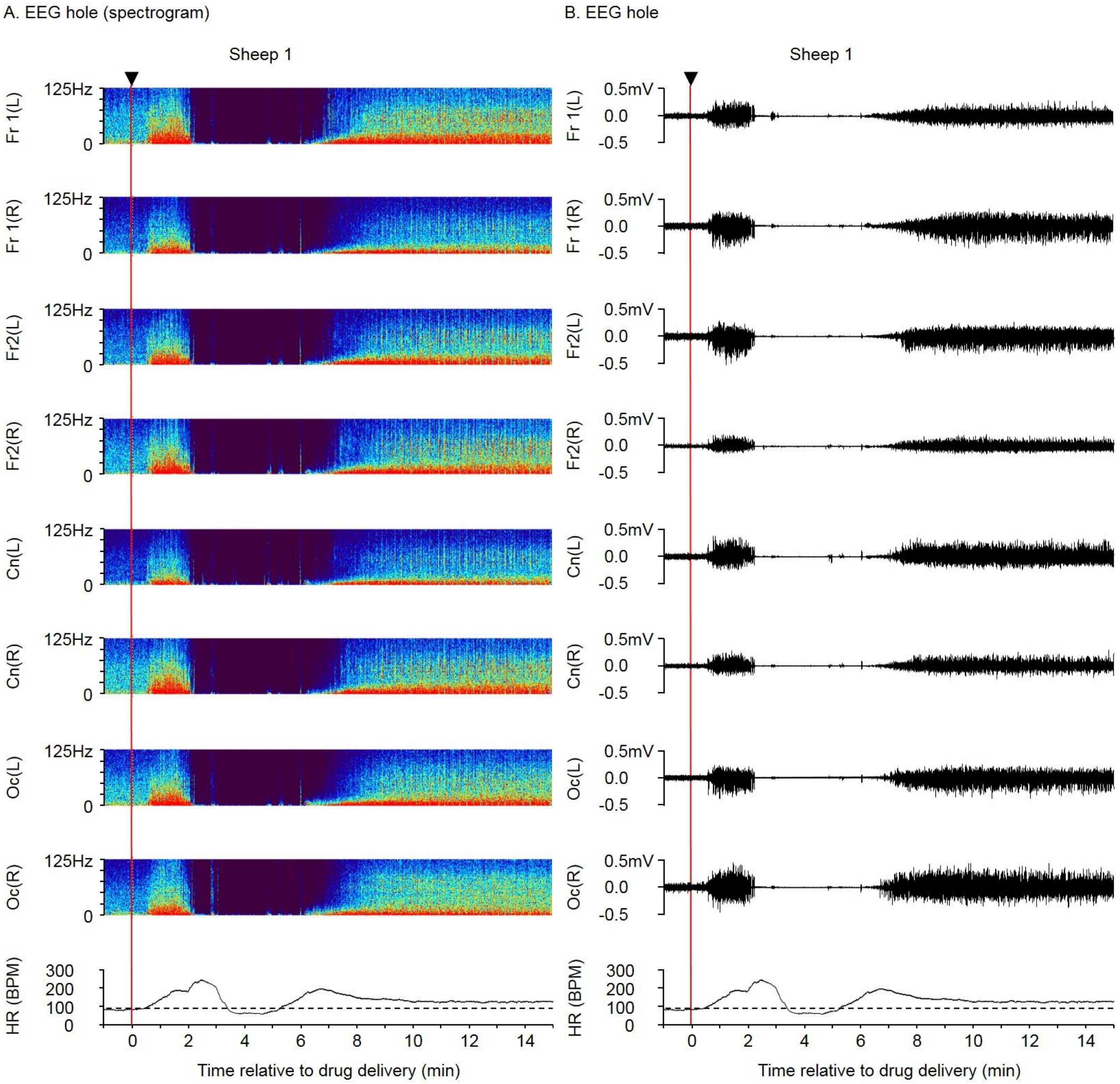

Supplementary Information Figure S3. The EEG 'hole' in response to the highest intravenous ketamine dose.

Spectrograms for all channels of EEG from a single sheep recorded following a 24mg/kg i.v. dose of ketamine (**A**). The corresponding EEG traces are shown for the same period in **B**. In each case delivery of intravenous ketamine at this dose resulted in shutdown of the cortical EEG across the brain, either completely or punctuated by intermittent, brief periods of activity. On recovery, the EEG resumed the characteristic alternation between low and high frequency oscillatory activity.

The sheep EEGs were recorded in two subgroups. The first EEG hole was seen in sheep 5 (Fig. 4E) that was recorded in Group 1. We did not record electrocardiogram (ECG) in this sheep. For the second group of sheep, when recordings were made at 24 mg/kg ketamine, we added an extra recording channel to collect ECG. In this example, the heart rate was initially elevated following drug administration, fell during the EEG hole and returning to an elevated rate as the EEG resumed.

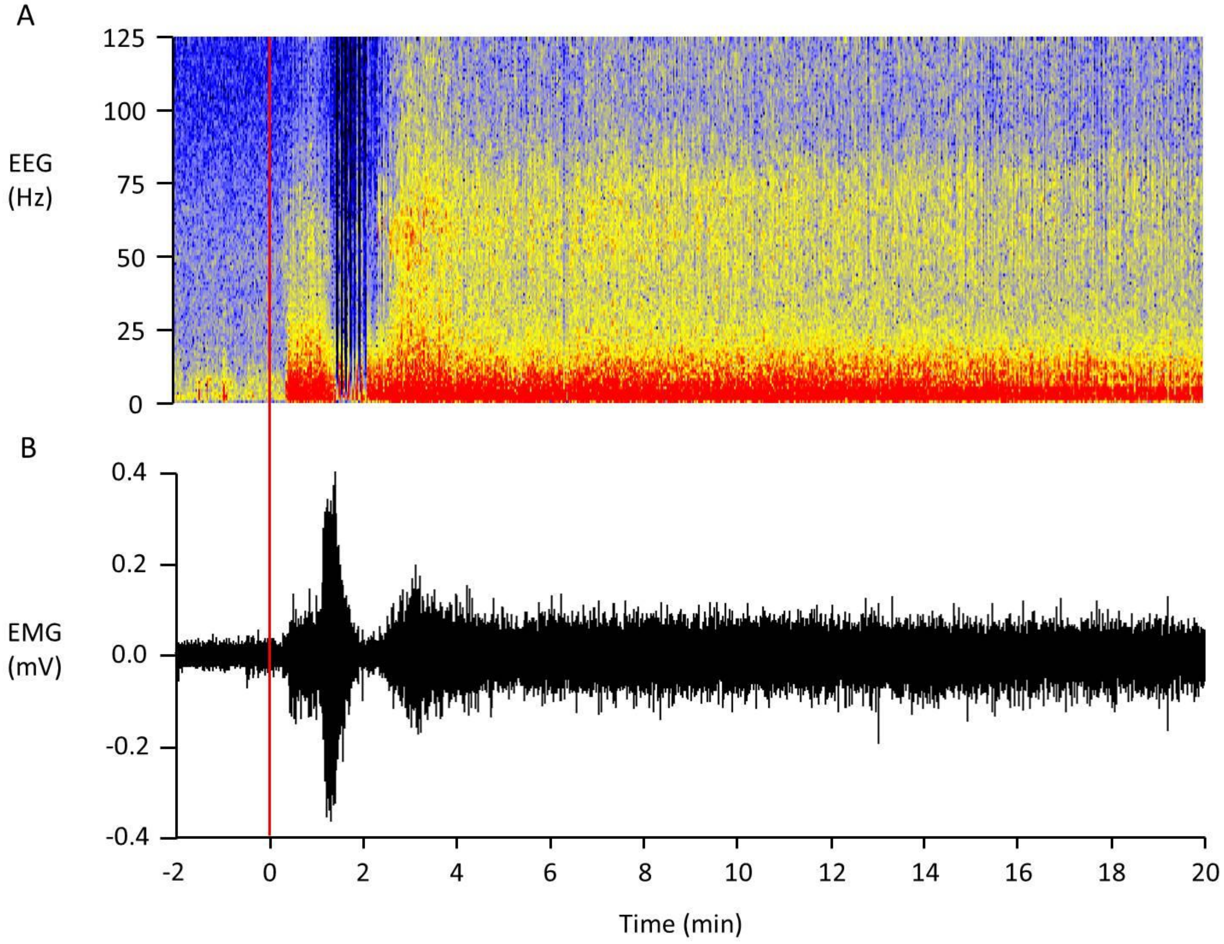

Supplementary Information Figure S4

(A) A spectrogram for a single EEG channel when 24mg/kg ketamine was delivered (red line) resulting in an EEG-hole, and (B) the simultaneous EMG recorded from the dorsal neck muscles. Delivery of ketamine causes increased EMG activity, which further increases just before the quiescent period in the EEG. During the EEG-hole, EMG activity declines to approximate pre-drug levels, and then increases as the EEG recovers its characteristic oscillatory response to the drug.

Supplementary Information Table S1.

Latency (s) to onset of EEG response to 12mg/kg ketamine.

		Latency (s)				
EEG Band	Route	Whole Brain	Frontal 1	Frontal 2	Central	Occipital
Full Spectrum (0 – 125Hz)	i.v.	31.7±0.7	30.9±1.1	32.0±1.3	31.8±1.7	32.3±1.6
	i.m.	109.5±5.0	102.8±11.2	113.4±13.3	110.2±11.8	113.3±13.3
Delta (0–4Hz)	i.v.	30.9±0.9	31.6±1.6	30.7±2.3	30.0±1.7	31.4±1.8
	i.m	113.0±5.6	113.0±13.5	115.6±12.7	112.7±36.7	111.7±14.5
Low Theta (4-6Hz)	i.v.	30.6±0.7	31.1±1.2	29.6±1.4	30.5±1.4	31.1±1.4
	i.m.	100.2±4.7	99.1±11.3	106.0±14.1	99.7±8.7	103.7±9.9
High Theta (6-9Hz)	i.v.	31.4±0.7	32.3±1.1	30.5±1.3	31.8±1.2	31.1±2.1
	i.m.	107.6±5.0	107.5±10.5	120.5±11.6	103.0±13.9	110.3±9.3
Alpha (9-14Hz)	i.v.	30.8±0.7	30.2±1.3	30.7±1.3	31.6±1.4	30.7±1.8
	i.m.	106.2±4.7	104.4±9.9	119.1±11.2	98.7±8.7	112.4±12.2
Beta (14-35Hz)	i.v.	34.0±0.7	33.0±1.5	32.9±1.3	35.5±1.5	34.5±1.6
	i.m.	129.5±6.2	114.7±13.2	131.6±10.8	127.2±13.4	136.6±18.3
Low Gamma (35-55Hz)	i.v.	39.3±0.9	39.6±1.3	39.9±1.5	39.6±1.9	40.0±2.1
	i.m.	150.8±7.6	125.0±4.5	120.0±3.7	147.0±10.7	187.5±24.3
Mid Gamma (55-85Hz)	i.v.	39.4±0.9	37.9±1.7	37.5±1.7	41.4±1.6	40.7±2.3
	i.m.	146.2±5.9	131.1±13.1	145.0±13.9	143.7±15.2	155.0±12.2
High Gamma (85-125Hz)	i.v.	47.9±2.5	48.2±2.0	47.5±2.5	48.9±2.5	47.0±2.4
	i.m.	167.0±6.1	161.2±15.4	166.4±11.2	167.6±6.0`	184.4±16.8

Supplementary Information Table S2.

Half-life (min) of EEG response to 12mg/kg ketamine.

		Half-life (min)				
EEG Band	Route	Whole Brain	Frontal 1	Frontal 2	Central	Occipital
Full Spectrum (0 – 125Hz)	i.v.	5.4±0.3	5.0±0.7	5.4±0.7	5.5±0.7	5.5±0.7
	i.m.	10.1±1.0	8.6±2.0	12.6±2.1	9.9±2.1	9.3±2.0
Delta (0–4Hz)	i.v.	4.2±0.3	3.6±0.7	3.9±0.7	5.3±0.7	3.9±0.7
	i.m.	7.5±1.1	6.2±2.0	9.2±2.3	7.8±2.1	6.8±2.1
Low Theta (4-6Hz)	i.v.	5.0±0.3	4.4±0.7	5.6±0.7	5.2±0.7	4.6±0.7
	i.m.	5.4±1.1	6.1±2.1	5.5±2.3	5.0±2.1	4.8±2.1
High Theta (6-9Hz)	i.v.	3.9±0.3	3.3±0.7	3.6±0.7	3.7±0.7	5.0±0.7
	i.m.	7.8±0.9	7.8±1.8	7.6±1.8	7.6±1.8	8.1±1.8
Alpha (9-14Hz)	i.v.	4.6±0.3	3.3±0.7	5.0±0.7	4.9±0.7	5.1±0.7
	i.m.	9.1±0.9	8.8±1.8	8.5±2.0	10.0±1.8	9.2±2.0
Beta (14-35Hz)	i.v.	5.0±0.3	4.4±0.7	4.7±0.7	5.8±0.7	4.9±0.7
	i.m.	13.1±0.9	15.7±1.8	11.1±2.0	11.5±2.0	14.0±1.8
Low Gamma (35-55Hz)	i.v.	4.8±0.3	5.8±0.7	5.1±0.7	3.5±0.7	4.8±0.7
	i.m.	16.2±1.0	18.8±2.0	14.4±2.1	17.2±2.0	14.4±2.3
Mid Gamma (55-85Hz)	i.v.	5.1±0.3	5.7±0.7	5.3±0.7	4.8±0.7	4.8±0.7
	i.m.	17.7±1.1	18.8±2.0	18.9±2.1	16.2±2.3	17.3±2.3
High Gamma (85-125Hz)	i.v.	6.1±0.3	4.9±0.7	6.3±0.7	6.3±0.7	6.9±0.7
	i.m.	17.9±1.0	18.4±1.8	18.0±2.0	18.2±1.8	17.1±2.2
